# Quercetin-induced apoptosis prevents EBV infection

**DOI:** 10.18632/oncotarget.3687

**Published:** 2015-03-29

**Authors:** Minjung Lee, Myoungki Son, Eunhyun Ryu, Yu Su Shin, Jong Gwang Kim, Byung Woog Kang, Gi-Ho Sung, Hyosun Cho, Hyojeung Kang

**Affiliations:** ^1^ College of Pharmacy, Institute of Microorganisms and Research Institute of Pharmaceutical Sciences, Kyungpook National University, Daegu, Republic of Korea; ^2^ Department of Medicinal Crop Research, National Institute of Horticultural and Herbal Science, Rural Development Administration, Eumseong, Republic of Korea; ^3^ Department of Oncology/Hematology, Kyungpook National University Medical Center, Kyungpook National University School of Medicine, Daegu, Republic of Korea; ^4^ Institute for Bio-Medical Convergence, International St. Mary's Hospital, College of Medicine, Catholic Kwangdong University, Incheon, Republic of Korea; ^5^ College of Pharmacy and Innovative Drug Center, Duksung Women's University, Seoul, Republic of Korea

**Keywords:** quercetin, isoliquiritigenin, Epstein-Barr virus, gastric carcinoma, apoptosis

## Abstract

Epstein-Barr virus (EBV) is a human gamma-1 herpesvirus that establishes a lifelong latency in over 90% of the world's population. During latency, virus exists predominantly as a chromatin-associated, multicopy episome in the nuclei of a variety of tumor cells derived from B cells, T cells, natural killer (NK) cells, and epithelial cells. Licorice is the root of *Glycyrrhiza uralensis* or *G. glabra* that has traditionally cultivated in eastern part of Asia. Licorice was reported to have anti-viral, anti-inflammatory, anti-atopic, hepatoprotective, anti-neurodegenerative, anti-tumor, anti-diabetic effects and so forth. Quercetin and isoliquiritigenin are produced from licorice and highly similar in molecular structure. They have diverse bioactive effects such as antiviral activity, anti-asthmatic activity, anti-cancer activity, anti-inflammation activity, monoamine-oxidase inhibitor, and etc. To determine anti-EBV and anti-EBVaGC (Epstein-Barr virus associated gastric carcinoma) effects of licorice, we investigated antitumor and antiviral effects of quercetin and isoliquiritigenin against EBVaGC. Although both quercetin and isoliquiritigenin are cytotoxic to SNU719 cells, quercetin induced more apoptosis in SNU719 cells than isoliquiritigenin, more completely eliminated DNMT1 and DNMT3A expressions than isoliquiritigenin, and more strongly affects the cell cycle progression of SNU719 than isoliquiritigenin. Both quercetin and isoliquiritigenin induce signal transductions to stimulate apoptosis, and induce EBV gene transcription. Quercetin enhances frequency of F promoter use, whereas isoliquiritigenin enhances frequency of Q promoter use. Quercetin reduces EBV latency, whereas isoliquiritigenin increases the latency. Quercetin increases more the EBV progeny production, and inhibits more EBV infection than isoliquiritigenin. These results indicate that quercetin could be a promising candidate for antiviral and antitumor agents against EBV and human gastric carcinoma.

## INTRODUCTION

Epstein-Barr virus (EBV) is a human gamma-1 herpesvirus that establishes a lifelong latency in over 90% of the world's population [1, 2]. During latency, the virus exists predominantly as a chromatin-associated, multicopy episome in the nuclei of a variety of tumor cells derived from B cells, T cells, natural killer (NK) cells, and epithelial cells [3]. Latent infection is associated with several malignancies, including Burkitt's lymphoma, Hodgkin's disease, post-transplant lymphoproliferative disease, NK/T-cell lymphoma, nasopharyngeal carcinoma, and gastric carcinoma [4, 5]. Average 10% of gastric carcinoma (GC) has been diagnosed as EBV associated GC (EBVaGC) [6]. In each EBV-positive cases of GC, EBV infects almost all gastric carcinoma cells [7]. It reported that the worldwide occurrence of EBVaGC is estimated at more than 50,000 cases per year [8]. Thus, EBVaGC is the most common cancer among EBV associated malignancies.

Licorice is the root of *Glycyrrhiza uralensis* or *G. glabra* that have traditionally been cultivated in eastern part of Asia. These plants are scientifically classified in *Glycyrrhiza* of *Fabales*. The word “licorice” is derived from the Greek meaning “sweet root” and demonstrated anti-viral, anti-inflammatory, anti-atopic, hepatoprotective, anti-neurodegenerative, anti-tumor, anti-diabetic effects and so forth [9]. *Glycyrrhiza* is known to produce a variety of bioactive compounds such as triterpene (glycyrrhizin, 18β(α)-glycyrrhetinic acid), isoflavan (glabridin, licoricidin), flavanone (liquiritin, liquiritigenin), chalconne (isoliquiritigenin, licochalcone A(B)), flavonol (quercetin), 3-arylcoumarin (glycyrol, glycyrin), and miscellaneous compounds [10].

Among these compounds, glycyrrhizic acid (GA) is a triterpene composed of one molecule of 18β-glycyrrhetinic acid and two molecules of D-glucuronic acid [11]. These component molecules of GA are released from upon hydrolysis. GA and its component compounds have exhibited antiviral effects against several viruses that include retrovirus, herpesvirus, influenza virus, hepatitis virus, enterovirus, and etc [12]. In particular, some herpesviral infection appeared to be inhibited by *in vitro* treatment of GA. Jung-Chung et al reported that early steps of EBV infection such as EBV attachment or penetration were interfered by GA treatment [13]. We previously showed that Kaposi's sarcoma associated herpesvirus (KSHV) latent infection was disrupted by GA treatment [14]. Physical binding of GA to cohesion resulted in loss of significant roles of CTCF-Cohesin complex on transcription of KSHV latent transcript unit. Hung et al found that GA perfusion in Herpes simplex virus (HSV) infection greatly decreased adhesion and stress between rat cerebral capillary vessel endothelial cells (CCECs) and polymorphonuclear leukocytes (PMN), suggesting that GA may attenuate inflammatory responses in HSV infection [15]. Therefore, GA is likely to be a major bioactive compound responsible for protective effects of licorice against viral infections. However, besides of GA, a variety of natural compounds has been isolated from licorice extracts. In order to precisely determine therapeutic effects of licorice, it is necessary to find out if these compounds also produce strong an antiviral effect like GA.

Based on molecular structures, flavonoids are classified into flavon, flavonol, flavanone, flavanol, isoflavone, chalcone, anthocyanin and catechin [16]. Quercetin and isoliquiritigenin are produced from licorice and highly similar in molecular structure [17]. Quercetin is a licorice flavonoid and its IUPAC name is 2-(3,4-dihydroxyphenyl)-3,5,7-trihydroxy-4H-chromen-4-one. Actually, quercetin belongs to a type of flavonols, which is a class of flavonoids that have the 3-hydroxyflavone backbone (3-hydroxy-2-phenylchromen-4-one) and present in a wide variety of herbs including licorice [10]. Isoliquiritigenin is classified into chalcone, which is an aromatic ketobe that forms a central core for chalcones or chalconoids [17]. It's IUPAC name is (*E*)-1-(2,4-dihydroxyphenyl)-3-(4-hydroxyphenyl)prop-2-en-1-one. Isoliquiritigenin exists in some foodstuffs and herbal medicines such as licorice [10].

Several studies demonstrated that quercetin had diverse bioactive effects such as antiviral activity, anti-asthmatic activity, anti-cancer activity, anti-inflammation activity, monoamine-oxidase inhibitor, and etc. Quercetin has shown to impair an oncoviral replication. Quercetin inhibited HBsAg and HBeAg secretion in the HBV (Hepatitis virus B)-producing 2.2.15 cells [18]. In addition, quercetin appeared to be the most effective inhibitor for HCV replication among all flavonoids, demonstrating a strong anti-HCV activity in HCV replicon-containing cells when combined with interferon (IFN)α [19]. Quercetin and its analog quercetin showed stronger inhibition on HIV-1 reverse transcriptase, all with IC_50_ values of 60 μM than HIV-1 protease and α-Glucosidase [20]. Quercetin was shown to inhibit other three reverse transcriptases from avian myeloblastosis, Rous-associated virus-2 and Maloney murine leukemia virus when poly(rA)oligo(dT)_12-18_ or rabbit globin mRNA were used as template [21]. Several studies investigated potential use for quercetin as anti-cancer agent [22]. A couple of cell culture studies showed that quercetin has an anti-cancer activity due to its antioxidant or anti-inflammatory properties [23]. Quercetin also inhibits the growth of cancer cells and helps induction of apoptosis [24]. Some studies using animal models have shown that quercetin could protect against colon cancer [25]. However, there is no reliable clinical evidence that quercetin has a protective effect on human cancer.

Isoliquiritigenin has reported to have various bioactive effects including antiviral activities, antioxidant activities, an antiplatelet aggregation effect, an antialdose reductase activity, estrogenic properties, and selective inhibition of H2 receptor-mediated signaling. Yuko et al found that *in vitro* Hepatitis C virus (HCV) replication was significantly suppressed by isoliquiritigenin and glycycoumarin, which were isolated from *Glycyrrhize radix* [26]. The suppression of HCV replication by two compounds appeared to be dose-dependent whose ED_50_s were 6.2 μg/ml and 15.5 μg/ml, respectively. Adianti et al discovered that isoliquiritigenin obtained from *Glycyrrhiza inflate* and *Glycyrrhiza glabra* showed anti-HCV activity, with IC_50_ of 3.7 μg/ml [11]. Therefore, we expect that both quercetin and isoliquiritigenin can be good therapeutic candidates for anti-EBV as well as EBV associated gastric cancer reagents.

To determine anti-EBV and anti-EBVaGC effects of licorice, we investigated first, antitumor effects of quercetin and isoliquiritigenin against EBVaGC, second, antiviral effects of quercetin and isoliquiritigenin against EBV, and third, the molecular mechanisms responsible for the antiviral and antitumor activities.

## RESULTS

### Both quercetin and isoliquiritigenin are cytotoxic to SNU719 cells

**Figure 1:**
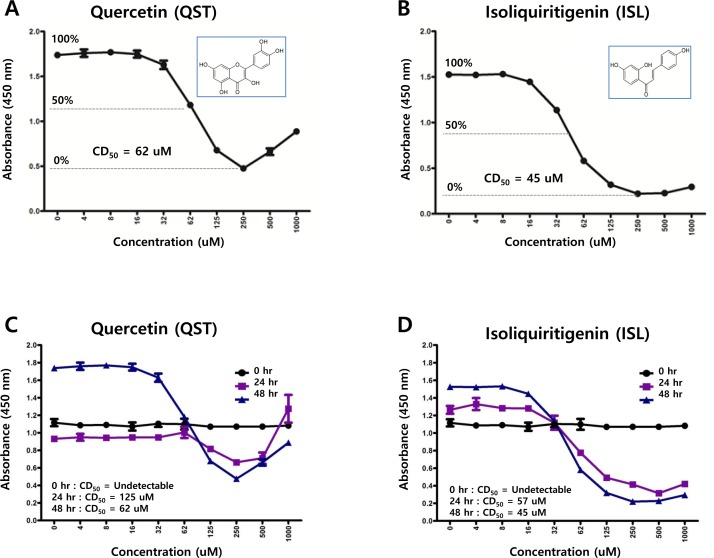

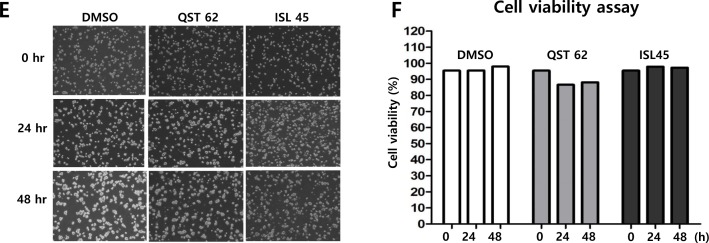
Structures of quercetin or isoliquiritigenin Cytotoxicity assay was conducted using cell counting assay (CCK-8 kit). (**A**, **B**) 50% cytotoxicity dose (CD_50_) of quercetin or isoliquiritigenin against SNU719 cells were 62 μM and 45 μM, respectively. Each measurement was repeated in three times. Averages and standard errors of measurements were displayed on graphs. ISL and QST stands for isoliquiritigenin and quercetin, respectively. Molecular structures of quercetin and isoliquiritigenin were defined inside Figure 1A and 1B. (**C**, **D**) Time kinetics of cytotoxicities of quercetin and isoliquritigenin, CD_50_s produced by treatments of quercetin or isoliquritigenin were determined on time course. (**E**, **F**) Time course cell viabilities of SNU719 cells treated with quercetin and isoliquiritigenin using cell viability assay using trypan blue staining. Cell viability was calculated as percentage of live cells relative to total cells. DMSO, QST62, and ISL45 stand for DMSO treatment, 62 μM isoliquiritigenin treatment, and 45 μM quercetin treatment, respectively.

As molecular structures of quercetin and isoliquiritigenin are similar each other, antitumor activities of quercetin was compared with those of isoliquiritigenin. In order to determine 50% cytotoxicity dose of quercetin or isoliquiritigenin against EBV associated gastric carcinoma cell line SNU719 cells, cellular cytotoxicity assay was conducted with Cell Counting Kit-8 (CCK-8) (Dojindo). CCK-8 allows for sensitive colorimetric assay determination of the number of viable cells in cell proliferation and cytotoxicity assays. 50% cytotoxicity dose (CD_50_) of quercetin and isoliquiritigenin against SNU719 were 62 μM and 45 μM, respectively (Figure 1A and 1B). In addition, in order to define time kinetics of cytotoxicities of quercetin and isoliquritigenin, CD_50_s produced by each compound treatment were determined on time course. During 48 h time course, CD_50_s were decreased from undetectable levels to values presented above (Figure 1C and 1D). Cell viability was also determined on time course in SNU719 cells treated with quercetin or isoliquiritigenin using cell viability assay using trypan blue staining. Although quercetin treatment showed slightly lower cell viability compared to the treatments of DMSO or isoliquritigenin, all treatments exhibited more than 85% of cell viability during the 48 h time course (Figure 1E and 1F).

### Quercetin induced more apoptosis than isoliquiritigenin, while both did not disrupt membrane integrity

**Figure 2:**
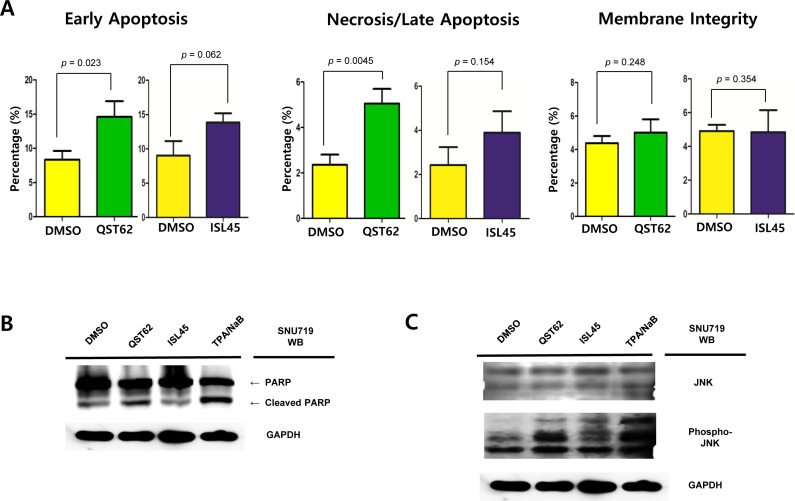

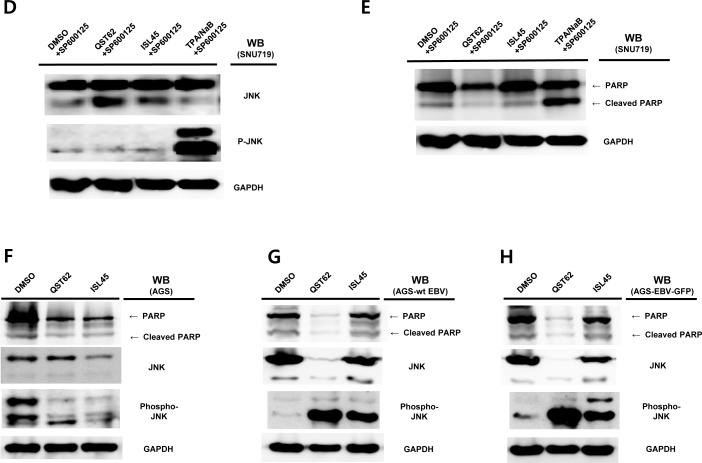
Effects of quercetin or isoliquiritigenin on apoptosis FITC Annexin V Apoptosis Detection assay and Western blot assay were conducted. (**A**) The effect of quercetin or isoliquiritigenin on early apoptosis (far left), necrosis/late apoptosis (middle) and membrane integrity (far right) in SNU719. DMSO, QST62, and ISL45 stand for DMSO treatment, 62 μM isoliquiritigenin treatment, and 45 μM quercetin treatment, respectively. Statistical significance is when the P-value is < 0.05 (95% confidence). (**B**, **C**) The effect of quercetin or isoliquiritigenin on the expression of PARP (**B**) and phospho-JNK (**C**). DMSO, QST62, and ISL45 stand for DMSO treatment, 62 μM isoliquiritigenin treatment, and 45 μM quercetin treatment, respectively. TPA/NaB is positive control. (**D**, **E**) The effect of co-treatment of SP600125 (10 μM, inhibitor of JNK kinase) with quercetin or isoliquiritigenin on the expression of phospho-JNK (**D**) and PARP (**E**). (**F**, **G**, **H**) The effect of EBV infection on the expression of cleaved PARP and Phospho-JNK in EBV-negative AGS cells (**F**), wild type (wt) EBV infected AGS cells (**G**), and recombinant EBV infected AGS cells (H) [41]. DMSO, QST62, ISL45, and TPA/NaB stand for DMSO treatment, 62 μM isoliquiritigenin treatment, 45 μM quercetin treatment, and TPA (20 ng/mL) and NaB (3 mM) co-treatment, respectively.

Two apoptosis assays as FITC Annexin V Apoptosis Detection assay and Western blot assay using anti-PARP antibody were conducted to analyze effects of quercetin or isoliquiritigenin on apoptosis and membrane integrity. First, FITC Annexin V Apoptosis detection assay was conducted to analyze cytotoxic effects on early apoptosis, necrosis/late apoptosis, and membrane integrity of SNU719 cells treated either quercetin or isoliquiritigenin for 48 h. Quercetin treatment induced significantly more early apoptosis and necrosis/late apoptosis in SNU719 cells than isoliquiritigenin treatment. Compared to DMSO treatment, quercetin treatment showed to induce early apoptosis approximately up to 175% and necrosis/late apoptosis up to 215%, whereas isoliquiritigenin treatment did not cause significant differences (Figure 2A, Supplemental Figure 1). However, membrane integrity of SNU719 cells was not affected by quercetin or isoliquiritigenin treatments, compared to DMSO treatment (Figure 2A, Supplemental Figure 1). These results indicated that quercetin and isoliquiritigenin did induce apoptosis without disrupting membrane integrity of SNU719 cells.

Western blot assay was conducted to determine if quercetin and isoliquiritigenin treatments cause any effect on PARP and cleavage PARP protein production in SNU719 cells. As PARP is known to help cells to maintain their viability [39], cleavage of PARP facilitates cellular disassembly and serves as a marker of cells undergoing apoptosis [39]. Compared of mock (DMSO), quercetin treatment showed significantly more cleaved PARP protein than isoliquiritigenin treatment (Figure 2B). In addition, as phosphatidyl JNK in JNK signal pathway is known to induces apoptosis [40], Western blot assay using anti-phospho-SAPK/JNK antibody was conducted to define if quercetin treatment enhances to produce phospho-SAPK/JNK. As expected, quercertin treatment increased phospho-SAPK/JNK production more than isoliquiritigenin treatment (Figure 2C). SP600125 is an anthrapyrazolone inhibitor of JNK kinase that competes with ATP to inhibit the phosphorylation of c-Jun [30]. In order to confirm that quercetin induces apoptosis through JNK pathway, SP600125 was co-treated in all treatments of licorice-derived compounds. The co-treatment of SP600125 (10 μM) suppressed the phosphorylation of c-Jun in all treatments except TPA/NaB treatment (Figure 2D). At the same time, SP600125 treatment significantly dampened quercetin mediated apoptosis, yet it produced mild effects on isoliquiritigenin mediated apoptosis (Figure 2E). Next we questioned if EBV infection plays an important role in quercetin mediated apoptosis. To answer this question, Western blot assay using PARP and Phospho JNK antibodies applied to three types of AGS cells such as EBV-negative AGS cells, wild type (wt) EBV infected AGS cells, and recombinant EBV infected AGS cells [41]. The JNK expression in EBV positive AGS cells was significantly higher than that in EBV negative AGS cells, while the phospho-JNK in EBV positive AGS cells was remarkably lower than that in EBV negative AGS cells (Figure 2F, 2G and 2H). However, the phosphorylation of JNK in EBV positive AGS cells was remarkably induced by treatments of quercetin and isoliquiritigenin, compared to that in EBV negative AGS cells (Figure 2F, 2G and, 2H). In detail, quercetin treatment showed slightly more phospho JNK form than isoliquiritigenin treatment in EBV positive AGS cells. Furthermore, the expression of PARP was completely inhibited by quercetin treatment in EBV positive AGS cells, yet that expression was not affected by quercetin treatment in EBV negative AGS cells. These results indicated that EBV infection is required for quercetin mediated apoptosis. Taken together, these studies led to suggest that quercetin is highly cytotoxic via apoptosis, which is mediated by JNK pathway and EBV infection.

### Isoliquiritigenin does not affect cell cycle, but quercetin strongly affects cell cycle

**Figure 3:**
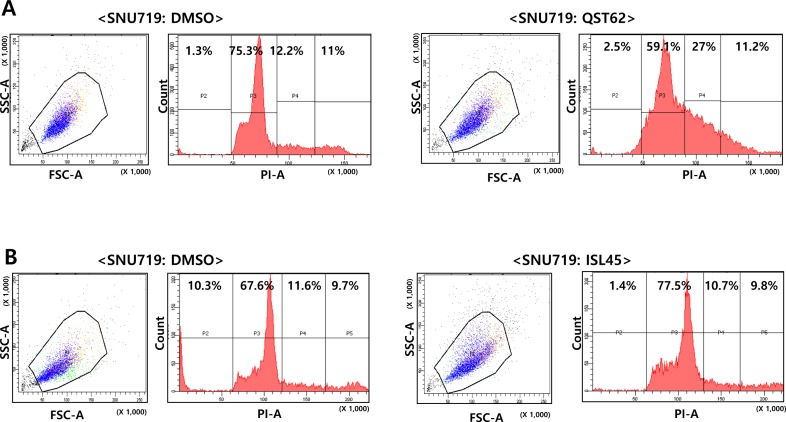
Effects of quercetin or isoliquiritigenin on cell cycle progress Effect of quercetin (62 μM, **A**) or isoliquiritigenin (45 μM, **B**) on cell cycle progress was determined using PI staining and FACs analysis. ISL and QST stands for isoliquiritigenin and quercetin, respectively.

To determine whether quercetin affected cell cycle progression in SNU719 cells, PI staining and FACs analysis were performed on SNU719 cells treated with quercetin or isoliquiritigenin for 48 h. Compared to mock treatment (DMSO), quercetin treatment significantly delayed S phase twice longer than mock treatment (Figure 3A), but isoliquiritigenin treatment slightly delayed G1 phase by 10% compared to mock treatment (Figure 3B). These results suggested that quercetin treatment did cause severe defects in normal cell-cycle progress by arresting S/G2 phase transition.

### Quercetin eliminated DNMT1 and DNMT3A expressions

**Figure 4:**
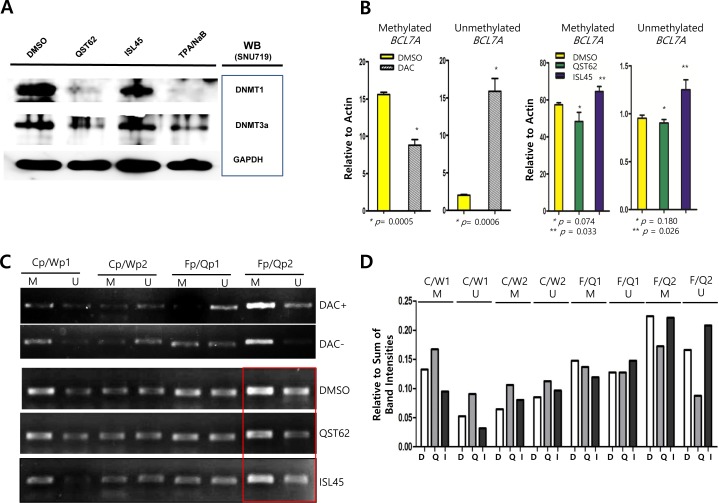

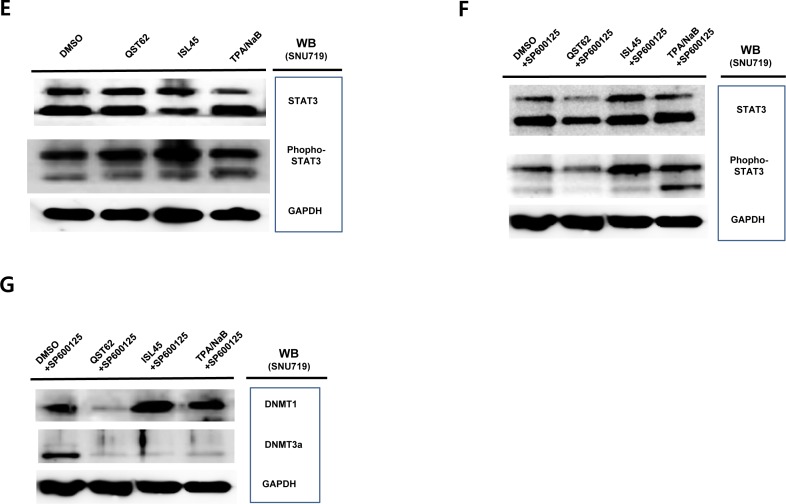
Effects of quercetin or isoliquiritigenin on methylation Western blot assay using anti-DNMTs and Stats antibodies, and methylation-specific PCR assay following RT-qPCR assay were conducted. (**A**) Western blot analysis using anti-DNMT1 and DNMT3A antibodies was conducted to determine if quercetin or isoliquiritigenin caused any effect on DNMT1 and DNMT3a protein production in the SNU719 cells. GAPDH was used loading and internal controls. (**B**) Methylation-specific PCR (MSP) assay was conducted to determine if treatments of quercetin or isoliquiritigenin affect methylation of *BCL7A*, tumor suppressor gene in the SNU719. As control, compared to DMSO treatment (negative control), 10 μM DAC (5-aza-2′-deoxycytidine) treatment suppressed methylation up to 35% and induced unmethylation up to 882% (left two panels). The MSP assay followed by RT-qPCR assay revealed that treatments of quercetin or isoliquiritigenin did not significantly affect methylation and unmethylation on *BCL7A* in SUN719 cells (right two panels). Statistical significance is when the P-value is < 0.05 (95% confidence). (**C**) The MSP assay followed by semiquantitative PCR assay was conducted to determine if treatments of quercetin or isoliquiritigenin affect methylation of EBV genomic locuses in SNU719 cells. Methylation on EBV genomic locus around Fp/Qp (Fp/Qp2 downstream region, in red box) was impacted by quercetin treatment, but not by isoliquiritigenin treatment. Cp/Wp1 and Cp/Wp2 stand for EBV genomic locuses of upstream and downstream regions of C/W promoters, respectively. Fp/Qp1 and Fp/Qp2 stand for EBV genomic locuses of upstream and downstream regions of F/Q promoters, respectively. M and U stand for methylation and unmethylation (demethylation) specific primers. DAC+ stands for treatment of 10 μM DAC (5-aza-2′-deoxycytidine) and DAC- stands for no treatment of DAC. (**D**) Resultant bands on agarose gels in Figure 3C were quantitated by densitometric analysis using ImageQuant 5.2 [35]. Intensities of all bands on agarose gel were summed to make a total band intensity. The intensity of each band was relatively evaluated compared to the total band intensity. (**E**) Western blot analysis using anti-STAT3 and anti-phospho-STAT3 antibodies was conducted to determine if quercetin or isoliquiritigenin caused any effect on STAT3 signaling pathways in the SNU719 cells. GAPDH was used loading and internal controls. (F, G) The effects of co-treatment of SP600125 (JNK inhibitor, 10 μM) with quercetin or isoliquritigenin on the phosphorylation of STAT3 (**F**) and the expression of DNMT1 and DNMT3a (**G**). DMSO, QST62, ISL45, and TPA/NaB stand for DMSO treatment, 62 μM isoliquiritigenin treatment, 45 μM quercetin treatment, and TPA (20 ng/mL) and NaB (3 mM) co-treatment, respectively.

In order to determine if quercetin or isoliquiritigenin affected methylation on CpG domains in cellular genes or EBV latent/lytic promoters, Western blot assay and methylation-specific PCR assays were performed. Western blot analysis using anti-DNMT1 (DNA methyltransferase 1) and DNMT3A (DNA methyltransferase 3A) antibodies revealed that quercetin treatment almost completely eliminated both DNMT1 and DNMT3A expression, while isoliquiritigenin treatment did not affect both DNMT1 and DNMT3A expressions (Figure 4A). Next, it was questioned if loss of DNMTs induced by quercetin might demethylate tumor suppressor genes and EBV locuses. Since SNU719 cells were known to densely methylated in *TP73*, *BLU*, *FSD1*, *BCL7A*, *MARK1*, *SCRN1*, and *NKX3.1* genes [42], methylation-specific PCR (MSP) following bisulfate conversion of DNA for methylation was conducted to define if quercetin and isoliquiritigenin affect methylation on tumor suppressor gene *BCL7A* and EBV promoter locuses. The MSP assay following realtime quantitative PCR revealed that compared to DMSO treatment, quercetin treatment slightly decreased methylation on CpG domain in *BCL7A* gene although it was statistically insignificant, while isoliquritigenin slightly increased the methylation (Figure 4B). Furthermore, the MSP assay following semi-quantitative PCR demonstrated that compared to DMSO treatment, quercetin treatment slightly decreased methylation on the downstream region of EBV F/Q promoter, while isoliquritigenin did not show any difference (Figure 4C). As controls in both MSP assay, compared to the mock treatment, 10 μM 5-aza-2′-deoxycytidine (DAC) treatment highly suppressed methylation and induced demethylation. When each band intensity was quantitated relative to sum of all band intensity using ImageQuant 5.2 [35], quercetin treatment showed decrease in both methylation and unmethylation around the downstream of Fp/Qp, while isoliquiritigenin treatment showed no significant effect on methylation and even slightly increased unmethylation around the downstream of Fp/Qp (Figure 4D).

Hino et al reported that STAT3 pathway is known to upregulate *DNMT1* [43]. Based on this report, it was questioned that qucercetin treatment might downregulate STAT3 or suppress phosphorylation of STAT3. To clarify this aim, western blot analysis using anti-STAT3 and anti-phospho-STAT3 antibodies was conducted. Compared to DMSO treatment, quercetin treatment did not suppress the phosphorylation of STAT3, while isoliquiritigenin treatment slightly enhanced the STAT3 phosphorylation. Unlike the previous study [43], this results indicated that quercetin downregulates DNMTs in a STAT3-independnt manner (Figure 4E). Moreover, co-treatment of SP600125 (JNK inhibitor, 10 μM) and quercetin clearly suppressed the phosphorylation of STAT3, while co-treatment of SP600125 and isoliquiritigenin remarkably enhanced the phosphorylation of STAT3 (Figure 4F). In the meantime, the co-treatment of SP600125 and quercetin totally abolished both DNMT1 and DNMT3 expressions, yet the co-treatment of SP600125 and isoliquiritigenin abolished only DNMT3a (Figure 4G). These results indicated that DNMT1 is regulated by quercetin in STAT3 independent manner and DNMT3 is controlled by quercetin in STAT3 dependent manner.

Taken together, this study suggested that reduction of demethylation by quercetin may play an important role in expression of hypermethylated genes in SNU719 genome and EBV genome.

### Both quercetin and isoliquiritigenin induce signal transductions to stimulate apoptosis

**Figure 5:**
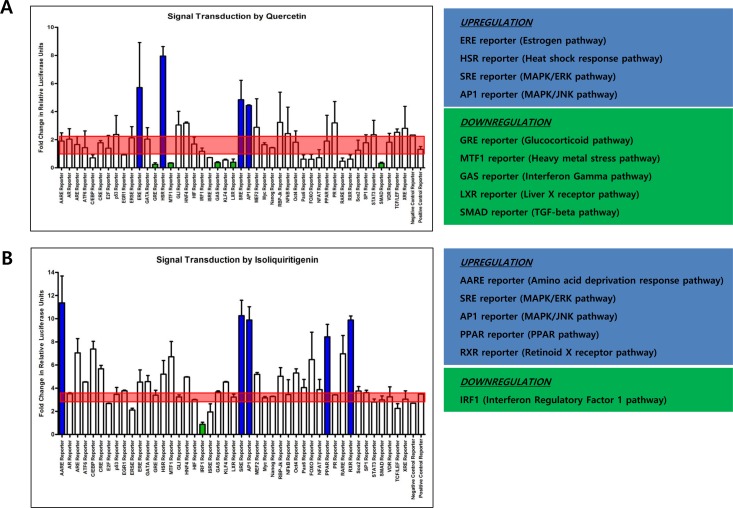
Effects of quercetin or isoliquiritigenin on signal transduction Cignal finder reporter assay was conducted to determine what signaling pathways inSNU719 cells are affected by treatments of quercetin or isoliquiritigenin. SNU719 cells were treated 62 μM quercetin or 45 μM isoliquiritigenin for 48 h and subjected to Cignal finder reporter assay. Each reporter is a luciferase construct in which a transcriptional factor specific to a signaling pathway was cloned. Signaling pathways affected by either quercetin or isoliquiritigenin were chosen when their fold changes were immensely higher or lower than negative and positive reporter signals (shaded by red box). Reporters induced by either quercetin or isoliquiritigenin were in blue box and reporters suppressed by either quercetin or isoliquiritigenin were in green box. ERE, HSR, SRE, AP1 reporters were upregulated by quercetin, and these reporters are specific to estrogen pathway, heat shock response pathway, MAPK/ERK pathway and MARK/JNK pathway, respectively. GRE, MTF1, GAS, LXR, SMAD reporters were downregulated by quercetin, and these reporters are specific to glucocorticoid pathway, heavy metal stress pathway, interferon gamma pathway, liver X receptor pathway and TGF-beta pathway, respectively. AARE, SRE, AP1, PPAR, RXR reporters were upregulated by isoliquiritigenin, and these reporters are specific to amino acid deprivation response pathway, MAPK/ERK pathway, MAPK/JNK pathway, PPAR pathway and retinoid X receptor pathway, respectively. IFR1 reporter was downregulated by isoliquiritigenin and this reporter is specific to interferon regulatory factor 1 pathway.

Since quercetin and isoliquiritigenin resulted in different expression patterns of DNMT1 and DNMT3A, it was considered that quercetin and isoliquiritigenin might go through different signaling pathways in SNU719 cells. Cignal finder reporter assay proved that quercetin treatment significantly stimulated 5 signaling pathways and suppressed 4 signaling pathways among 45 signaling pathways tested (Figure 5A). The 4 activated signaling pathways were estrogen, heat shock response, MAPK/ERK, and MAPK/JNK pathway. The 5 suppressed signaling pathways were glucocorticoid, heavy metal stress, interferon gamma, liver X receptor, and TGF-beta pathway. On the other hand, isoliquiritigenin treatment remarkably induced 5 signaling pathways and repressed one signaling pathway (Figure 5B). The 5 activated signaling pathways are amino acid deprivation response, MAPK/ERK, MAPK/JNK, PPAR, and retinoid X receptor pathway. The suppressed signaling pathway was interferon regulatory factor 1 pathway. Signaling pathways regulated by either quercetin or isoliquiritigenin is simultaneously involved in both cellular apoptosis and survival programs. For example, the activation of MAPK/JNK pathway by both quercetin and sioliquiritigenin treatments supported the induction of apoptosis by quercetin and isoliquiritigenin because JNK pathway is likely to proceed downstream signaling for apoptosis [40].

In addition, downregulation of glucocorticoid pathway by quercetin could support the induction of apoptosis by quercetin because glucocorticoid is known to trans-repress AP-1 that transduces signals for apoptosis [44]. Downregulation of interferon regulatory factor 1 pathway (IRF1) by isoliquiritigenin could result in the reduction of apoptosis because IRF1 is known to induce apoptosis [45].

### Both quercetin and isoliquiritigenin induce EBV gene transcription

**Figure 6:**
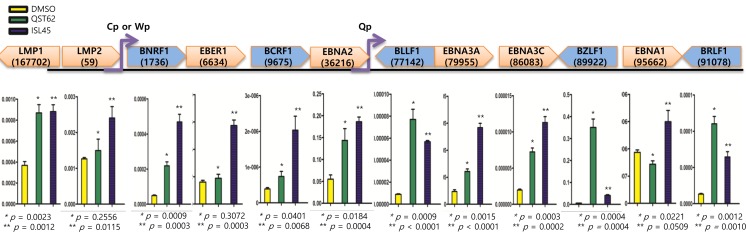
Effects of quercetin or isoliquiritigenin on EBV latent and lytic transcription RT-qPCR assay was conducted to define if treatments of quercetin or isoliquiritigenin affect transcriptions of EBV latent and lytic genes in SNU719 cells. cDNA synthesized from RNAs in SNU719 cells treated 62 μM quercetin or 45 μM isoliquiritigenin were subjected to RT-qPCR assay. Compared to DMSO treatment (negative control), quercetin significantly increased transcriptions of lytic genes such as *BNRF1*, *BCRF1*, *BLLF1*, *BZLF1* and *BRLF1*, and it increased also transcriptions of latent gene such as *LMP1*, *EBNA3A* and *EBNA3C*. Isoliquiritigenin significantly increased transcriptions of lytic genes such as *BNRF1*, *BCRF1*, *BLLF1*, *BZLF1* and *BRLF1*, and it increased also transcriptions of latent gene such as *LMP1*, *LMP2*, *EBER1*, *EBNA3A*, *EBNA3C* and *EBNA1*. Statistical significance is when the P-value is < 0.05 (95% confidence). ISL and QST stands for isoliquiritigenin and quercetin, respectively.

To find if quercetin or isoluqiritgenin has an antiviral effect on EBV transcription, we investigated transcription patterns in SNU719 cells treated with either quercetin or isoliquiritigenin using RT-qPCR assay (Figure 6). Compared to mock treatment, quercetin treatment significantly increased transcription of lytic genes such as *BNRF1*, *BCRF1*, *BLLF1*, *BZLF1* and *BRLF1* and latent genes such as *LMP1, EBNA3A,* and *EBNA3C*. Isoliquiritigenin treatment enhanced transcription of lytic genes such as *BNRF1*, *BCRF1*, *BLLF1*, *BZLF1*, and *BRLF1* and latent genes such as *LMP1*, *LMP2*, *EBER1*, *EBNA3C* and *EBNA1*. However, compared to isoliquiritigenin treatment, quercetin treatment upregulated more EBV lytic genes such as *BLLF1*, *BZLF1*, and *BRLF1*. On the other hand, compared to quercetin treatment, isoliquiritigenin treatment upregulated EBV latent genes such as *LMP2*, *EBER1*, *EBNA3A*, *EBNA3C*, and *EBNA1*. Given that *BZLF1*, key factor for EBV lytic reactivation, was upregulated by quercetin and *EBNA1*, key factor for EBV latency establishment, was upregulated by isoliquritigenin [46], these results indicated that quercetin stimulates EBV lytic replication cycle while isoliquiritigenin stimulates EBV latent replication cycle.

### Quercetin enhances EBV lytic proteins, while isoliquiritigenin enhances EBV latent proteins

**Figure 7:**
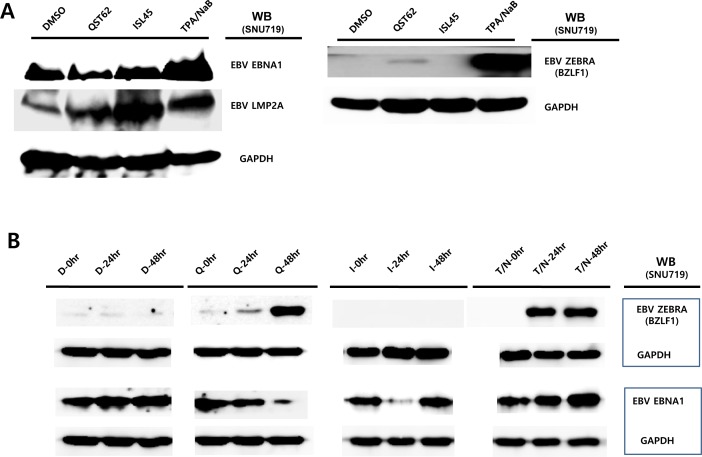

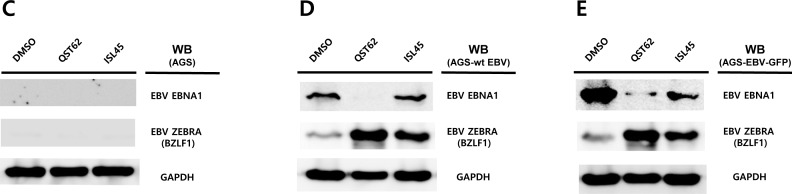
Effects of quercetin or isoliquiritigenin on EBV protein production EBV Protein levels were assessed by Western blot assay using anti-EBV EBNA1, BZLF1 and LMP2A antibodies to define if treatments of quercetin or isoliquiritigenin affect EBV translation in SNU719 or AGS cells. (**A**) The effects of treatments of quercetin or isoliquritigenin on EBV protein expression in SNU719 cells. Treatments were conducted for 48 h. GAPDH was used as an internal control in the western blot analysis. ISL and QST stands for isoliquiritigenin (62 μM) and quercetin (45 μM), respectively. (**B**) Time kinetics of effects of quercetin and isoliquritigenin on EBV protein production in SNU719 cells. Treatments were conducted for 0 h, 24 h and 48 h. D, Q, I and T/N stands for treatments of DMSO, quercetin (45 μM), isoliquritigenin (62 μM) and TPA (20 ng/mL) /NaB (3 mM), respectively. (C, D, E) The effect of EBV infection on the expression of EBV protein expression in EBV-negative AGS cells (**C**), wild type (wt) EBV infected AGS cells (**D**), and recombinant EBV infected AGS cells (**E**) [41]. DMSO, QST62, ISL45, and TPA/NaB stand for DMSO treatment, 62 μM isoliquiritigenin treatment, 45 μM quercetin treatment, and TPA (20 ng/mL) and NaB (3 mM) co-treatment, respectively.

Similar to the transcription assay, we were interested in whether the expression of EBV protein was also affected by quercetin or isoliquiritigenin treatments. To analyze EBV protein expression, Western blot assay using anti-EBV EBNA1, BZLF1, and LMP2A antibodies was conducted with SNU719 treated either quercetin or isoliquiritigenin for 48 h. Like transcription patterns of EBV genes affected by both compounds, the Western assay showed that quercetin did enhance BZLF1 and LMP2A expressions, however, isoliquiritigenin remarkably induced LMP2A expressions (Figure 7A). In addition, in order to define time kinetics of effects of quercetin and isoliquritigenin on EBV protein production, the effects by the two compounds were determined on time course of SNU719 treated with quercetin or isoliquritigenin. During 48 h time course, quercetin treatment enhanced BZLF1 expression, yet suppressed EBNA1 expression. In contrast, isoliquiritigenin treatment exhibited little effect on BZLF1 expression and EBNA1 expression during the time course except 24 h post treatment (Figure 7B).

Next we questioned if the EBV lytic gene is induced by quercetin in different EBV associated gastric carcinoma cells. To answer this aim, western blot assay using EBNA1 and BZLF1 antibodies applied to three types of AGS cells such as EBV-negative AGS cells, wild type (wt) EBV infected AGS cells, and recombinant EBV infected AGS cells [41]. BZLF1 expression was not detected in EBV negative AGS cells, yet the expression was significantly induced by quercetin or isoliquiritigenin in EBV positive AGS cells (Figure 7C, 7D and, 7E). In detail, quercetin treatment induced slightly more BZLF1 than isoliquiritigenin. In contrast, the expression of EBNA1 in EBV positive AGS cells was not induced by isoliquiritigenin treatment and it was even severely suppressed by quercetin treatment, compared to that in EBV negative AGS cells (Figure 7C, 7D and 7E). These results indicated that the BZLF1 induction by quercetin is a common event in EBV associated gastric carcinoma cells. Taken together, these studies led to suggest that quercetin is likely to induce EBV lytic reactivation and isoliquiritigenin tends to maintain EBV latent replication in EBV associated gastric carcinoma cells.

### Quercetin enhances frequency of F promoter use, whereas isoliquiritigenin enhances frequency of Q promoter use

**Figure 8:**
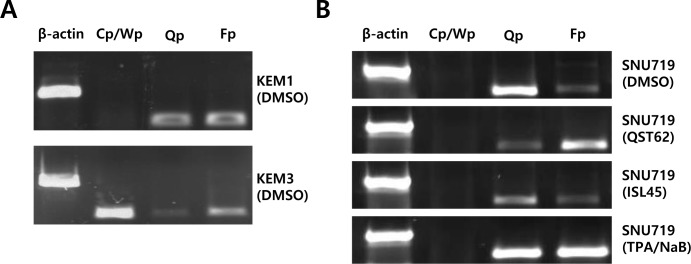
Effects of quercetin or isoliquiritigenin on frequency of use of EBV promoters Reverse transcription followed PCR (RT-PCR) assay was conducted to determine if treatments of quercertin or isoliquiritigenin affect on frequency of use of EBV promoters in SNU719 cells. (**A**) KEM1 control cells specific to EBV type I showed high frequency of use of Qp and Fp. KEM3 control cells specific to EBV type III demonstrate high frequency of use of Cp/Wp. (**B**) Treatment of quercetin (62 μM) intensively increased frequency of use of EBV Fp, compared to that of EBV Qp in SNU719 cells. However, treatment of isoliquiritigenin (45 μM) intensively increased frequency of use of EBV Q promoter, compared to that of EBV Fp promoter in SNU719 cells. Cp, Wp, Qp, and Fp stand for EBV promoters activated depending on EBV latency types. NaB/TPA treatment stands for 1 mM treatment of NaB and 1 ng/ml treatment of TPA. ISL and QST stands for isoliquiritigenin and quercetin, respectively.

**Figure 9:**
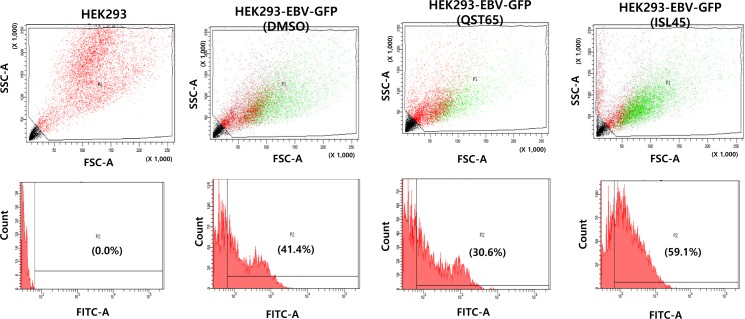
Effects of quercetin or isoliquiritigenin on EBV latency maintenance *In vitro* HEK293 cell & EBV-GFP infection assay followed FACS analysis was conducted to determine if treatments of quercetin or isoliquiritigenin affect maintenance of EBV latency in SNU719 cells. Measurement of the FITC emission from FACS Aria III helps determine the cytometric profile of EBV latency in HEK293-EBV-GFP cells. HEK293-EBV-GFP cells are HEK293 cells that were latently infected EBV recombinant viruses and selected with hygromycin B. Compared to DMSO treatment (negative control), quercetin treatment (62 μM) decreased FITC emission up to 11%, but isoliquiritigenin treatment (45 μM) increased FITC emission up to 18%. HEK293 cells were subjected to FACS analysis as an internal negative control. ISL and QST stands for isoliquiritigenin and quercetin, respectively.

Because both quercetin and isoliquiritigenin affected expression of most EBV genes, it was questioned whether quercetin or isoliquiritigenin affected the frequency of use of EBV latency promoters such as Cp, Qp, and Fp [2]. As controls, KEM1 cells (EBV latency type 1-positive cells) showed a high frequency of use of Q and F promoters (Figure 9A) [47]. KEM3 cells specific to EBV type III demonstrated high frequency of use of C/W promoters (Figure 8A) [47]. Quercetin treatment significantly decreased frequency of use of Q promoter and increased frequency of use of F promoter, supporting functional role of quercetin for EBV lytic reactivation (Figure 8B). By contrast, isoliquiritigenin treatment clearly decreased frequency of use of F promoter and increased frequency of use of Q promoter, supporting functional role of isoliquiritigenin for EBV latency establishment (Figure 8B).

### Quercetin reduces EBV latency, whereas isoliquiritigenin increases the latency

To further confirm different roles of quercetin and isoliquiritigenin in EBV life cycle, HEK293 cells based EBV *in vitro* infection system was applied to determine how quercetin or isoliquiritigenin affect EBV latency [31]. HEK293-EBV-GFP cells are derived from HEK293 cells by infecting with EBV recombinant virus. Measurement of the FITC emission from FACS Aria III helps determine the cytometric profile of EBV latency in HEK293-EBV-GFP cells treated with either quercetin or isoliquiritigenin (Figure 9). The assay demonstrated that quercetin treatment decreased FITC emission up to 11%, suggesting weakness of EBV latent replication, and isoliquiritigenin increased FITC emission up to 18%, suggesting reinforcement of EBV latent replication. The results obtained from HEK293 cell system also supported the different roles of quercetin or isoliquiritigenin on EBV life cycles.

### Quercetin more increases the EBV progeny production than isoliquiritigenin

**Figure 10:**
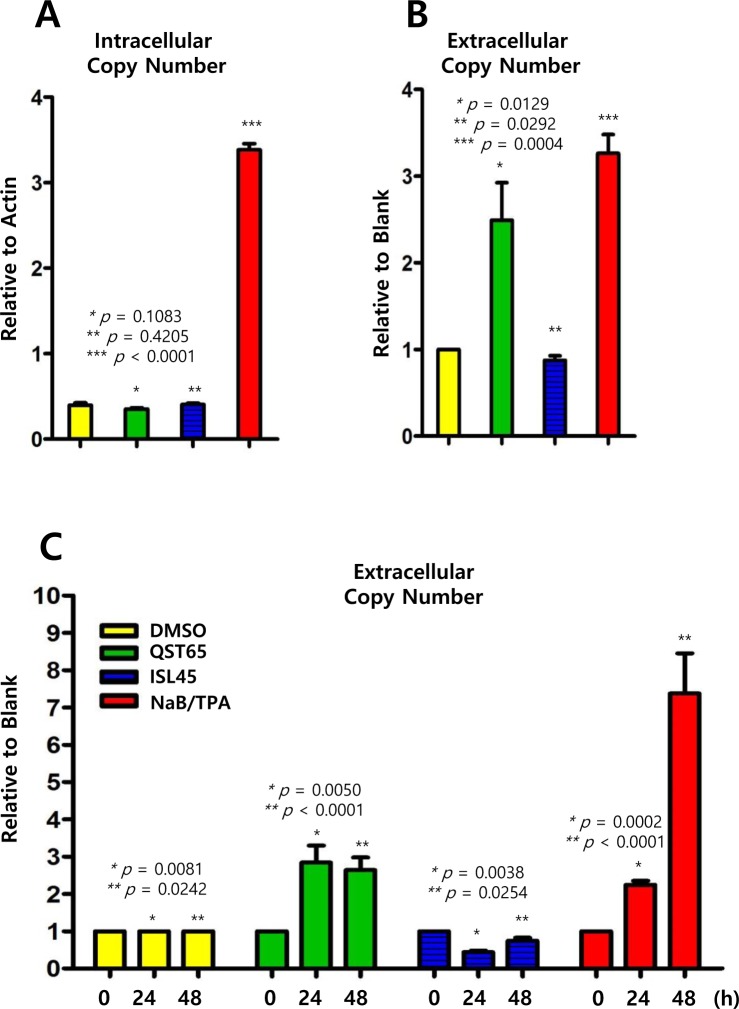
Effects of quercetin or isoliquiritigenin on EBV progeny production Intracellular and extracellular EBV genome copy numbers in SNU719 cells were determined by following methods previously described to evaluate antiviral effects of quercetin and isoliquritingenin on EBV progeny production. (**A**) Compared to DMSO treatment (negative control), EBV intracellular copy numbers were not changed by treatments of quercetin (62 μM) or isoliquiritigenin (45 μM). (**B**) Compared to DMSO treatment (negative control), EBV extracellular copy numbers were remarkably increased by quercetin treatment, while the copy numers were not affected by isoliquiritigenin. Intracellular and extracellular copy numbers were calculated as relative intracellular (relative to Actin) and extracellular (relative to blank treatment) EBV genome copy numbers, respectively. Statistical significance is when the P-value is < 0.05 (95% confidence). NaB/TPA treatment stands for 1 mM treatment of NaB and 1 ng/ml treatment of TPA. ISL and QST stands for isoliquiritigenin and quercetin, respectively. (**C**) Time kinetics of effects of quercetin and isoliquritigenin on EBV progeny production in SNU719 cells. During 48 h time course, quercetin treatment showed to significantly enhance EBV progency production since 24 h post treatment. DMSO, QST62, ISL45, and TPA/NaB stand for DMSO treatment, 62 μM isoliquiritigenin treatment, 45 μM quercetin treatment, and TPA (20 ng/mL) and NaB (3 mM) co-treatment, respectively.

Because most EBV genes tested were regulated by quercetin or isoliquiritigenin, we next asked whether these two compounds stimulated EBV progeny production. Therefore, EBV intracellular and extracellular genome copy numbers were evaluated using previous described methods [14, 29]. Intracellular EBV genome copy numbers were not affected by treatments with either quercetin or isoliquiritigenin (Figure 10A), but extracellular EBV genome copy numbers were significantly increased up to 150% by quercetin treatment (Figure 10B). In addition, in order to define time kinetics of effects of quercetin or isoliquritigenin on EBV progeny production, the effects by two compounds were determined on time course of SNU719 cells treated with quercetin or isoliquritigenin. During 48 h time course, quercetin treatment significantly enhanced EBV progency production since 24 h post treatment. In contrast, isoliquiritigenin treatment exhibited almost no effects on EBV progeny production during the time course (Figure 10C). These results indicated that quercetin is likely to derive EBV lytic replication and subsequently to produce EBV progenies from SNU719 cells.

### Quercetin more inhibits EBV infection than isoliquiritigenin

**Figure 11:**
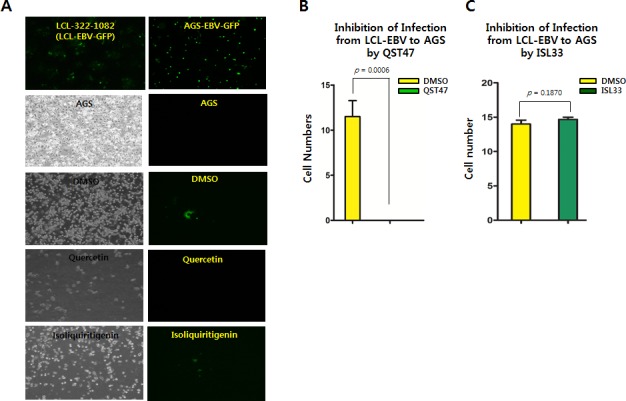
Effects of quercetin or isoliquiritigenin on EBV infection Cell to cell coinfection assay using LCL-EBV and AGS cells was conducted to determine if treatments of quercetin or isoliquiritigenin affect EBV infection. (**A**) EBV could transfer from LCL-EBV cells to AGS cells using cell to cell coculture infection system. AGS cells infected EBV-GFP virus were in attached form and GFP florescent. (**B**) As a donor, LCL-EBV-GFP cells (LCL-2266-36) was cocultured with AGS cells seeded previously. CD_50_s of quercetin and isoliquiritigenin against AGS cells were determined as 62 and 58 μM, respectively. These CD_50_ values, 62 μM quercetin and 45 μM isoliquiritigenin were applied to define their effect on EBV infection. Compared of DMSO treatment (negative control), quercetin intensively suppressed the transfer of EBV from LCL-EBV-GFP cells to AGS cells, meaning inhibition of EBV infection to gastric epithelial cells. (**C**) However, isoliquiritigenin treatment did not cause any effect on the EBV horizontal infection. Statistical significance is when the P-value is < 0.05 (95% confidence). ISL and QST stands for isoliquiritigenin and quercetin, respectively.

It was also questioned whether quercetin or isoliquiritigenin have effects on preventing EBV infection to virus-susceptible cells. LCL-EBV-GFP [37] and AGS [38] cell to cell coinfection assay was designed to test if quercetin or isoliquiritigenin treatments could favor EBV infection from LCL to AGS cells (Figure 11A). EBV could be transferred from LCL-EBV to AGS using co-culture infection system. Compared to mock (DMSO), quercetin treatment significantly suppressed the transfer of EBV from LCL-EBV-GFP cells to AGS cells, but isoliquiritigenin treatment did not have any effect on EBV infection (Figure 11B). We postulated that EBV progenies produced by quercetin treatment failed to infect quercetin-treated AGS cells, and yet EBV progenies produced by isoliquiritigenin succeeded to infect isoliquiritigenin-treated AGS cells. These results suggested that quercetin treatment is likely to cause severe defects on EBV infection steps such as EBV receptor recognition and EBV entry.

## DISCUSSION

In this study, we showed that both quercetin and isoliquiritigenin isolated from licorice have distinguished antitumor or antiviral activities against gastric carcinoma and EBV. Given our observations, antitumor and antiviral effects of quercetin and isoliquiritigenin were followed. CD_50_ values of quercetin and isoliquiritigenin were 62 μM and 45 μM against SNU719 cells, respectively (Figure 1). Quercetin induced a strong early apoptosis and necrosis/late apoptosis in SNU719 cells (Figure 2). Quercetin significantly arrested S/G2 transition of SNU719 cells, whereas isoliquiritigenin did not impact on the cell cycle progress (Figure 3). Quercetin showed demethylation in cellular and viral genomes (Figure 4). Quercetin and isoliquiritigenin appeared to induce signal transductions linked to apoptosis, such as MAPK/JNK pathway and MAPK/ERK pathway (Figure 5). Quercetin upregulated noticeably EBV lytic genes such as *BZLF1*, *BRLF1* and *BLLF1*, while isoliquiritigenin upregulated markedly EBV latent genes such as *LMP1*, *LMP2*, *EBNA3A* and *EBNA1* (Figure 6). Quercetin enhanced to produce BZLF1, yet isoliquiritigenin remarkably induced to produce LMP2A (Figure 7). Frequency of EBV promoter use was affected by compounds isolated from licorice. Quercetin enhanced Fp frequency than Qp, but isoliquiritigenin preferred Qp to Fp (Figure 8). EBV latency was weakened by quercetin, but it was reinforced by isoliquiritigenin (Figure 9). Only quercetin stimulated to produce EBV progeny viruses from SNU719 cells (Figure 10). Interestingly, infection of EBV from lymphocyte to gastric adenocarcinoma cells was severely inhibited by quercetin, while the infection was not affected by isoliquiritigenin (Figure 11).

Quercetin is a naturally occurring flavonoid and isoliquiritigenin is a flavonoid phytoestrogen from licorice. Both compounds are highly similar in molecular structures like analogs; 2-(3,4-dihydroxyphenyl)-3,5,7-trihydroxy-4*H*-chromen-4-one for quercertin, and (*E*)-1-(2,4-dihydroxyphenyl)-3-(4-hydroxyphenyl)prop-2-en-1-one for isoliquiritigenin (Figure 1A). Thus they were expected to exhibit similar effect on cancer and oncoviral infection. Certainly, both quercetin and isoliquiritigenin are known to induce apoptosis in a variety of human cancer cells [48-53]. Both compounds showed to downregulate anti-apoptic factors such as Bcl-2, Bcl-xL, and Mcl-1 and cleavage caspase-9, caspase-3 and PARP. They also demonstrated to downregulate cell-cycle essential factors such as CDK2, CDK6, cyclin D, cyclin E and cyclin A. Several mechanisms were reported to trigger the apoptosis caused by treatment of quercetin and isoliquritignin. One is to induce apoptosis by blocking of PI3K/AKT pathway that tends to upregulate anti-apoptotic factors [19, 53]. Another is to induce apoptosis by arresting cell cycle at G0, G1, and G2 phases [54, 55]. The other is to cause apoptosis by activating endoplasmic reticulum stress-signaling pathway [56, 57]. The last is to cause apoptosis by activating JNK/ERK pathway [48, 58]. However, there might be other mechanisms to induce apoptosis. Based on our study, quercetin was likely to induce apoptosis through JNK/ERK pathway.

SNU719 cells used in our study were derived from EBV associated gastric carcinoma, and thus EBV is latently infected in the SNU719 cells. Quercetin and isoliquiritigenin showed remarkably different signaling patterns on inducing apoptosis in SNU719 cells at 50% cytotoxic dose (CD_50_). Quercetin produced clearer cleaved PARP and stronger activation of JNK pathway than isoliquiritigenin, even though both compounds caused to lose cell membrane asymmetry and integrity. Thus, it was concluded that isoliquiritigenin did not cause significant apoptosis from SNU719 cells.

There could be at least three factors that cause the different effects on apoptosis. First, it could be that isoliquiritigenin does not work as ligands for TNF-R1 to induce apoptosis from SNU719 cells. Second, as the genome of SNU719 cell is known to heavily methylated [42], it could be so methylated most signaling pathways including JNK/ERK pathway that these pathways might be silenced by methylation on CpG islands of transcriptional factors essential to apoptosis induction. This methylation mediated silencing may be a reason why isoliquiritigenin did not induce apoptosis from SNU719 cells. Indeed, quercetin showed to reduce DNMTs expression through downregulating of STAT3 signaling pathway and consequently decrease methylation on Fp/Qp region (Figure 4). Thus, it is possible that some hypermethylated genes essential for apoptosis induction may be demethylated and active for gene expression by quercetin treatment.

Third, in some points of views, signaling pathways could to support different roles of quercetin or isoliquiritigenin on cellular apoptosis. Activation of MAPK/JNK pathway by both quercetin and isoliquiritigenin supported the induction of necrosis/late apoptosis by quercetin or isoliquiritigenin because JNK pathway is tightly linked to apoptosis induction pathway [40]. In addition, downregulation of glucocorticoid pathway by quercetin could support the induction of apoptosis by quercertin because glucocorticoid is known to trans-repress AP-1 that transduces signals for apoptosis [44]. Downregulation of interferon regulatory factor 1 pathway (IRF1) by isoliquiritigenin could result in the reduction of apoptosis by isoliquiritigenin because IRF1 is known to induce apoptosis [45]. Taken together, our study clearly showed different effects of quercetin and isoliquiritigenin on inducing apoptosis in SNU719 cells. Furthermore, we can't rule out possibility that isoliquiritigenin does not induce apoptosis in SNU719 cells in which quercetin causes strong apoptosis. Fourth, given that both compounds can induce similar apoptosis is in most cancer cells as reported by many previous studies [41-46], we could speculate that EBV latently infected SNU719 cells might play a pivotal role in inducing apoptosis by quercetin. As one of speculations, EBV is known to produce anti-apoptotic factors that possibly contribute to evade apoptosis of EBV infected host cells [59]. Isoliquiritigenin might downregulate these anti-apoptotic EBV genes that subsequently produce weaker apoptosis of SNU719 cells, compared to quercetin. It is definitely necessary to dissect further downstream mechanisms for apoptosis by both compounds.

Like to antitumor effects, it was observed different antiviral effects between quercetin and isoliquiritigenin [11, 13, 15, 26]. Consistent with previous studies, our study also observed antiviral effects of quercetin and isoliquiritigenin. In particular, quercetin plays an important role in producing EBV progeny viruses from SNU719 cells, while isoliquiritigenin plays a clear role in maintaining EBV latency in SNU719 cells. Indeed, quercetin treatment led to produce a large quantity of EBV progeny viruses in SNU719 cells, and then it raised abrupt release of many EBV particles from host cells. This abrupt release of a large quantity of EBV progeny virus might contribute to cause to lyse host cell plasma membrane. This lysis might trigger next level of apoptosis which isoliquiritigenin can't cause. However, these speculations should be tested in further studies to get them more convinced.

Interestingly, quercetin almost completely prevented EBV from infecting neighbor AGS cells, while isoliquiritigenin did not inhibit EBV to infect the AGS cells. Quercetin seemed to be a potent apoptosis inducing agents at 50% cytotoxicity dose against AGS cells. Apoptosis is likely to losing cell membrane asymmetry that impacts to disrupt adequate receptors for EBV entry such as CD21, CD35 and HLAII [2]. Ultimately, loss of EBV receptors on AGS cells resulted in the loss of EBV infection from LCL to AGS cells.

Both quercetin and isolquritigenin were reported to induce cell apoptosis through cell cycle arrest in various human-derived tumor cells. The inhibitory effect of quercetin or isolquritigenin on cell cycle progression seems to be mediated by down-regulation of cyclins and Cdk proteins [53, 60, 61]. Interestingly, our study shows that quercetin has much stronger effect on cell cycle arrest than isolquiritigenin (Figure 3). In fact, the effect of quercetin on the expression of EBV-derived proteins is little investigated. We found that quercetin could stimulate the lytic induction of EBV through up-regulated expression of *BLLF1*, *BZLF1* and *BRLF1*, which is shown in Figure 6. Kudoh et al. reported that the induction of EBV lytic activation completely arrested cell cycle progression in p21, p53-independent way [62]. Moreover, the cell cycle arrest by quercetin has been reported to associate with the induction of apoptosis as well as prevention of liver carcinogenesis [55]. A couple of other studies also described the correlation between EBV lytic cycle activation and cell cycle arrest [29]. Therefore, we postulate that quercetin inhibits cell cycle progression much more vigorously than isoliquiritigenin because of direct activation of quercetin on EBV lytic replication, which is not seen by isoliquiritigenin.

Based on all findings in this study, a working model for molecular mechanism used by quercetin and isoliquiritigenin for antitumor and antiviral activities could be suggested. Quercetin downregulates the expression of DNMTs in a STAT3-independent manner. Due to the lack of DNMTs, demethylation occurs on SNU719 and EBV genome. These demethyated locuses may include CpG motifs in SNU719 *JNK* and EBV *BZLF1* genes. Upon being demethylated by quercetin treatment, *JNK* and *BZLF1* genes tend to be active and play key roles in inducing apoptosis and EBV lytic replication. On the other hand, isoliquiritigenin failed to downregulate DNMTs in SNU719 cells. Thus a plenty of DNMTs expression would lead to maintain anti-apoptotic capacity of SNU719 cells and EBV latency.

Roots from licorice are most popularly used as pharmacological sources for treatment of certain disorders [10]. This is because the root is known to contain rich reserves of biologically effective compounds such as glycyrrhizic acid, Glycyrrhetinic acid, lincoricidin, isoliquiritigenin, quercetin and etc [12]. Our study tested if quercetin or isoliquiritigenin were effective for antiviral and antitumor activities and determined that quercetin is intensively potent to antiviral effects through apoptosis. Thus, licorice roots containing quercetin can be used to protect host cells from EBV infection and EBV associated disorders. In terms of developing licorice as medicinal food, one of future studies must focus on determining if licorice roots are clinically effective in protecting cancer development or EBV infection from patients. This novel study highlights a fundamental possibility of the development of licorice roots as a medicinal food in order to prevent EBV and gastric carcinoma.

## MATERIALS AND METHODS

### Preparation of quercetin and isoliquiritigenin

Quercetin and isoliquiritigenin were obtained from Sigma-Aldrich Co. (St. Louis, MO, USA; catalog number I3766 and Q4951). Isoliquiritigenin and quercetin were dissolved in DMSO to make stock solution of 200 mM, which was then filtered through 0.22-μm filter (Sartorius Stedim Biotech) and stored at −20°C until use.

### Cell cultures and reagent

SNU719 cells, a gastric carcinoma cell line that contains EBV genome as episome, were cultured in RPMI1640 (Welgene, Daegu, Korea) supplemented with 10% fetal bovine serum (FBS, Welgene), antibiotics/antimycotics (Gibco, Grand Island, NY, USA), and Glutamax (Gibco) at 37°C, 5% CO_2_, and 95% humidity in a CO_2_ incubator [27]. HEK293-EBV-GFP cells are HEK293 cells which EBV latently infected. HEK293 and HEK293-EBV-GFP were cultured in Dulbecco's Modified Eagle Medium (DMEM, Cellgrom, USA) supplemented with 10% Fetal Bovine Serum (Omega, Canada), antibiotics/antimycotics (Gibco, USA), and Glutamax (Gibco) at 37°C, 5% CO2, 95% humidity in a CO2 incubator [28]. HEK293-EBV-GFP were selected with hygromycin B (400 μg/ml). AGS cells are EBV negative gastric adenocarcinoma cells. AGS were cultured in RPMI1640 supplemented with 10% Fetal Bovine Serum (Omega, Canada), antibiotics/antimycotics (Gibco, USA), and Glutamax (Gibco) at 37°C, 5% CO2, 95% humidity in a CO2 incubator [28, 29]. AGS-wt EBV cells and AGS-EBV-GFP cells were gifts from Dr. Cho in KRIBB and Dr. Lee in Catholic University of Korea. AGS-EBV-GFP cells were selected with G418 (400 μg/ml). For inhibitor studies, the C-Jun N-terminal Kinase (JNK) inhibitor, SP600125 was purchased Sigma-Aldrich (St. Louis, MO, USA) and dissolved in DMSO (dimethyl sulfoxide) [30].

### Reverse-transcription quantitative polymerase chain reaction

RNA was extracted from either 62 μM quercetin or 45 μM isoliquiritigenin-treated SNU719 cells by using an RNeasy Mini Kit (Qiagen, Valenica, CA, USA), and was then synthesized into cDNA by using Superscript II Reverse Transcriptase (Invitrogen). The resultant cDNA was diluted 1:50 in nuclease-free water and used to analyze expression of EBV latent and lytic genes by quantitative polymerase chain reaction (qPCR). Primers for the following EBV latent genes were used: *LMP2A*, *EBER*, *EBNA3A*, and *EBNA1*. Primers for the following EBV lytic genes were used: *BNRF1*, *BLLF1*, *BZLF1*, *BRLF1*, and *BNLF2A*. Internal control gene was *Actin*. All primer set sequences have been published previously [31], and are available upon request. Positive controls used in these experiments were HDAC inhibitors such as sodium butyrate (3 mM) and 12-O-tetradecanoylphorbol-13-acetate (TPA, 20 ng/mL). qPCR was performed using iQ SYBR Green reagent (Bio-Rad) in qPCR CFX96 (Bio-Rad). Compared to mock treatment, sample treated either quercetin or isoliquiritigenin was analyzed for EBV gene expression in triplicate.

### Intracellular and extracellular EBV genomic DNA copy number quantification

Following lysis and sonication using a Bioruptor sonicator (Cosmobio, Tokyo, Japan; 5 min, 30-s on/off pulses), genomic DNA (gDNA) was extracted from either 62 μM quercetin or 45 μM isoliquiritigenin for different time courses. Each resultant gDNA (50 ng) was subjected to qPCR analysis, and the relative amount of EBV gDNA was determined using the internal control *Actin*. Intracellular EBV copy number was calculated as the relative amount of EBV gDNA in the total gDNA. To determine the relative extracellular EBV copy number, 20-ml culture medium samples were collected from SNU719 cells treated with either quercetin or isoliquiritigenin. The culture medium samples were filtered through a 0.45-nm syringe filter, loaded onto a 20% sucrose cushion in phosphate-buffered saline (PBS) solution, and subjected to ultracentrifugation (CP100WX, Hitachi) at 27,000 rpm for 90 min. The virus pellet was lysed in 100 μl of FA lysis buffer [EDTA (1 mM, pH 8.0), HEPES-KOH (50 mM, pH 7.5), and NaCl (140 mM)], sonicated using a Bioruptor sonicator for 5 min (30-s on/off pulses), and DNA was extracted. Finally, viral DNA was resuspended in 100 μl of RNase-free water and qPCR analysis was used to quantify viral DNAs by using primer sets specific for EBV *EBNA1*.

### Cell cycle analysis

To assess if quercetin or isoliquiritigenin affect cell cycle progression in SNU719 cells, they were treated with quercetin and isoliquiritigenin, stained with propidium iodide (PI) solution for 48 h, and then subjected to cell cycle analysis by using a FACSAria III cell sorter (BD Bioscience; San Jose, CA, USA). Briefly, 3 × 10^6^ cells were seeded in 60-mm culture dishes and cultured overnight. On the following day, cells were treated with 62 μM quercetin or 45 μM isoliquiritigenin. The cells were harvested using trypsin at 48 h post-treatment, washed with cold PBS, fixed in 95% ethanol for at least 1 h, treated with 300 μg of RNase A, stained in 10× PI solution, and finally analyzed for cell cycle progress by using a FACSAria III cell sorter. Dimethyl sulfoxide (DMSO) was used as mock in each treatment.

### Cytotoxicity assay

To evaluate the cytotoxic effects of quercetin or isoliquiritigenin on SNU719 cells, a cellular cytotoxicity assay was performed using Cell Counting Kit-8 (CCK-8 ; Dojindo, Kumamoto, Japan) [32]. Briefly, 100 μl of cell suspension (1 × 10^4^ cells/well) was seeded and then treated with various concentrations (0-1000 μM) of quercetin or isoliquiritigenin. After 0 h, 24 h and 48 h of quercetin or isoliquiritigenin treatment, 10 μl of CCK-8 solution was added to each sample. Samples were incubated for another 3 h, and the absorbance of each cell suspension was then measured using an enzyme-linked immunosorbent assay reader. All steps followed the manufacturer's recommended protocol. In addition, to determine on time course cell viabilities, cell counting assay using trypan blue staining was conducted. Trypan blue is one of several stains recommended for use in dye exclusion procedures for viable cell counting. This assay is based on the principle that live (viable) cells do not take up certain dyes, whereas dead (non-viable) cells do [33]. Cell viability was calculated as percentage of live cells relative to total cells.

### Western blot analysis

To assess if quercetin or isoliquiritigenin impact on EBV protein synthesis, Western blotting was performed in SNU719 or AGS cells treated with 62 μM quercetin or 45 μM isoliquiritigenin. Treated SNU719 cells were harvested using trypsin in 0 h, 24 h and 48 h post treatment. Cells (10 × 10^6^) were then lysed using 100 μl of reporter lysis buffer (Promega, Madison, WI, USA) supplemented with 1 μl of proteinase inhibitor and 10 μl of phenylmethylsulfonyl fluoride. The cell lysates were further fractionated using the Bioruptor sonicator (5 min, 30-s on/off pulses). When necessary, the cell lysates were snap frozen in liquid nitrogen and stored at −80°C. Cell lysates (25 μl) were loaded onto 7% sodium dodecyl sulfate polyacrylamide electrophoresis gel and subjected to Western blot analysis by using antibodies against EBV proteins (1:1000 dilution). Following antibodies were used: anti-EBV EBNA1 (Santa Cruz Biotechnology, Dallas, TX, USA), anti-EBV BZLF1(Santa Cruz Biotechnology), anti-EBV LMP2A(Santa Cruz Biotechnology), anti-DNMT1(Santa Cruz Biotechnology), anti-DNMT3A(Santa Cruz Biotechnology), anti-PARP(Cell Signaling Technology, Beverly, MA, USA), anti-SAPK/JNK(Cell Signaling Technology), anti-phospho-SAPK/JNK(Cell Signaling Technology), anti-beta-actin (Santa Cruz Biotechnology), anti-GAPDH(Cell Signaling Technology), anti-STAT3 (Cell Signaling Technology), and anti-phospho-Stat3(Cell Signaling Technology). Horseradish peroxidase-conjugated sheep anti-mouse IgG (Amersham Biosiences, Glattbrugg, Switzerland), horseradish peroxidase-conjugated donkey anti-rabbit IgG (Amersham Biosiences), and horseradish peroxidase-conjugated goat anti-rat IgG (Bethyl Laboratories, Montgomery, TX, USA) were used as secondary antibodies.

### Apoptosis and membrane integrity analysis

To determine whether quercetin or isoliquiritigenin induced apoptosis and membrane integrity of SNU719 cells, an Annexin V-FITC apoptosis detection assay was conducted. Briefly, 5 ml of SNU719 cells (1 × 10^6^) was seeded in a 6-cm plate, and treated with 62 μM quercetin or 45 μM isoliquiritigenin (CD_50_ value) on the following day. After 48 h of treatment, cells were stained using FITC Annexin V Apoptosis Detection Kit I (BD Pharmingen, San Jose, CA, USA), and were then analyzed early apoptosis, necrosis/late apoptosis, and membrane integrity of SNU719 cells using a FACSAria III cell sorter 1 h post-staining.

### Methylation-specific PCR

To determine if quercetin or isoliquiritigenin affect tumor suppressor gene methylation in SNU719 cells, a methylation-specific PCR assay was performed using DNA subjected to bisulfite conversion as described previously [34]. Following lysis and sonication by using a Bioruptor sonicator (5 min, 30 s on/off pulses), gDNA was extracted from SNU719 cells treated with 62 μM quercetin or 45 μM isoliquiritigenin. We used the CpGenome DNA Modification Kit (Millipore, Billerica, MA, USA) for sodium bisulphite conversion of the DNA. The sequences of the *BCL7A* primers used are shown in Table 1. Each 25-μl reaction contained 5 μl of bisulfite-treated DNA template, 5 μl of 5× reaction mix (NanoHelix, Daejeon, Korea), 5 μl of 5× TuneUp solution (NanoHelix), 1 μl of Taq-plus polymerase (NanoHelix), and 2.5 μl of 10 μM forward/reverse primer. Primers were specific for methylated and unmethylated *BCL7A*. The primer pairs specific for regions upstream and downstream of C and W promoters (Cp/Wp) as well as upstream and downstream of F and Q promoters (Fp/Qp) are listed in Table 1. The following cycle conditions were used: 95°C for 3 min; 30 cycles of 95°C for 30 s, 55°C for 30 s, and 72°C for 30 s; followed by 72°C for 10 min. The reactions were performed using a TaKaRa PCR Thermal Cycler (Otsu, Japan) and then run on a 1.5% agarose/TBE gel. Resultant bands on agarose gels were quantitated by densitometric analysis using ImageQuant 5.2 [35]. Intensities of all bands on agarose gel were summed to make total band intensity. The intensity of each band was relatively evaluated compared to the total band intensity.

**Table 1:** Primers for methylation-specific & EBV promoter usage polymerase chain reaction

Genomic DNA		Primer sequence (5′- 3′)		Primer sequence (5′ - 3′)
*BCL7A*	MF	GGTAGGCGACGTTTTAGTTC	UF	TGGGGTAGGTGATGTTTTAGTTT
	MR	GAATTAAAAACACCGATTCG	UR	CCAAATTAAAAACACCAATTCAA
Upstream of EBV Cp/Qpregion	MF(11147-11259)	TTTAAAGTGOTAATAATATTAGGCGG	UF	TTAAAGTGGTAATAATATTAGGTGG
	MR(11148-11259)	CTACATTTTTCAAATCGTAAACGAA	UR	CTACATTTTIVAAATCATAAACAAA
Downstream of EBV Cp/Wpregion	MF(18971-19084)	GTTTTTTAGAGGAATTAGGGATTTTG	UF	TTTTTTAGAGGAATTAGGGATITC
	MR(18972-19087)	TCAAACATTCTTTAAATTTAACGAA	UR	CCCICAAACATTCTITAAATRTAACA
Upstream of EBV Fp/Qpregion	MF(45005-45106)	TTTGGGGTATGGTATATTTAGTAGC	UF	TGGGGTATGGTATATTTAGTAGTGT
	MR(45007-45107	AACCTAATTCTTAACTCGTTCGAC	UR	AAACCTAATTC7TAACTCATTCAAC
Downstream of EBV Fp/Qpregion	MF(53255-53371)	ATTGTTTTATTTAGTTGGTGGTGTC	UF	TGTTTTATTTAGTTGGTGGTGTTGA
	F(36134-36152)	CAAAATTTCCTAAC T T T TTACGAA	UR	ACAAAATTTCCTAACTTTTTACAAA
EBV Cp/Wp	F(50152-50168)	TGCCTGAACCTGTGGTTGG	R(95664-95654/55361-55348)	CATGATTCACACTTAAAGGAGACGG
EBV Qp	F(50152-50168)	GTGCGCTACCGGATGGC	(95664-95654/55361-55348)	CATGATTCACACTTAAAGGAGACGG
EBV Fp	F(50099-50115)	GGGTGAGGCCACGCTTT	R(55326-55304)	CAGGTCTAC7GGCCTGTCTATGAT

### Promoter usage detection assay

To determine if quercetin or isoliquiritigenin affect the selection of EBV promoter usage, we performed conventional PCR on cDNA isolated from SNU719 cells treated with 62 μM quercetin or 45 μM isoliquiritigenin. Total RNA was extracted from SNU719 cells treated with quercetin or isoliquiritigenin by using an RNeasy Mini Kit (Qiagen), and RNA was then synthesized into cDNA using Superscript II Reverse Transcriptase. As controls, cDNA was synthesized from total RNA collected from KEM1 and KEM3 cells, which are EBV latency type 1 and 3 Burkitt lymphoma cells, respectively [36]. Primer sequences, including those for actin, EBV Qp, EBV Cp/Wp, and EBV Fp, were published previously [31] and listed in Table 1. cDNA was amplified in 25-μl reactions containing 5 μl of 5× reaction mix, 5 μl of 5× TuneUp solution, 1 μl of Taq-plus polymerase, and 2.5 μl of 10 μM of forward/reverse primer. The following cycle conditions were used: 95°C for 3 min; 30 cycles of 95°C for 10 s, 55°C for 30 s, and 72°C for 10 min; followed by 72°C for 10 min. The reactions were performed using a TaKaRa PCR Thermal Cycler and then run on a 1.5% agarose/TBE gel.

### Gammaherpesvirus infection

To determine whether quercetin or isoliquiritigenin affect EBV infection of AGS cells (gastric carcinoma), we performed cell-to-cell coinfection by using AGS and LCL-EBV-GFP B-cell lymphocyte cells. LCL-EBV-GFP B-cell lymphocyte cells were obtained from Lieberman's lab [37]. AGS cells [38] (0.625 × 10^6^/well) were seeded in 6-well plates (Corning, NY, USA). The following day, AGS cell culture medium-RPMI1640 (Hyclone, Logan, UT, USA) supplemented with 10% FBS (Hyclone), antibiotics/antimycotics, and Glutamax-was replaced with fresh medium, AGS cells were overlaid with LCL-EBV-GFP cells (1.25 × 10^6^/ml), and were treated with 62 μM quercetin or 58 μM isoliquiritigenin for 72 h. CD_50_s of quercetion and isoliquiritigenin against AGS cells were determined as 62 and 58 μM, respectively. These CD_50_ values were applied to this EBV infection assay using AGS cells. 72 hours post-coinfection, we completely removed the medium and washed the cells twice with PBS, leaving only AGS-EBV-GFP cells. Bacto agar (1.5%) containing 2× RPMI1640 (Hyclone) supplemented with 20% FBS (Hyclone), antibiotics/antimycotics, and Glutamax was applied to the infected AGS-EBV-GFP cells. After 72 h of incubation, we counted GFP foci formed on AGS cells treated with either compounds or control treatment (Dimethyl sulfoxide, DMSO).

### Cignal finder reporter array

To determine what SNU719 cell signaling pathways were affected by quercetin or isoliquiritigenin treatment, a Cignal Finder Multi-Pathway Reporter Array (Qiagen, catalog number CCA-901L) was used. Attractene (1 μl/well) was distributed into a 96-well Cignal Finder Multi-Pathway Reporter Array plate. SNU719 cell suspension was diluted in Opti-MEM medium and then seeded in each well. Cells were treated with either 62 μM quercetin or 45 μM isoliquiritigenin on the following day. After 48 h of treatment, reverse transfection reagent and Opti-MEM medium were removed. Dual-Glo Luciferase Reagent (75 μl) was then added to each well and plates were incubated for 10 min at room temperature. Finally, firefly and renilla luciferase activities were measured following the manufacturer's recommendations. Briefly, 75 μl of firefly luciferase reagent was added to each well and then luciferase activity was measured. Afterwards, 75 μl of Dual-Glo Stop&Glo luciferase reagent was added to each well and renilla luciferase activity was measured.

### Statistical analysis

Statistical tests were performed using unpaired t-test and ANOVA. P-values (one-tailed) <0.05 (95% confidence) were considered statistically significant.

## SUPPLEMENTARY MATERIAL AND FIGURE


