# Supplementary material for: Quercetin-induced apoptosis prevents EBV infection
Source: Oncotarget. 2015 Mar 29;6(14):12603–24. doi: 10.18632/oncotarget.3687 (PMC4494961; doi:10.18632/oncotarget.3687)
Supplement: Supplementary file 1 [file oncotarget-06-12603-s001.pdf]

Quercetin-induced apoptosis prevents EBV infection

Supplementary Material

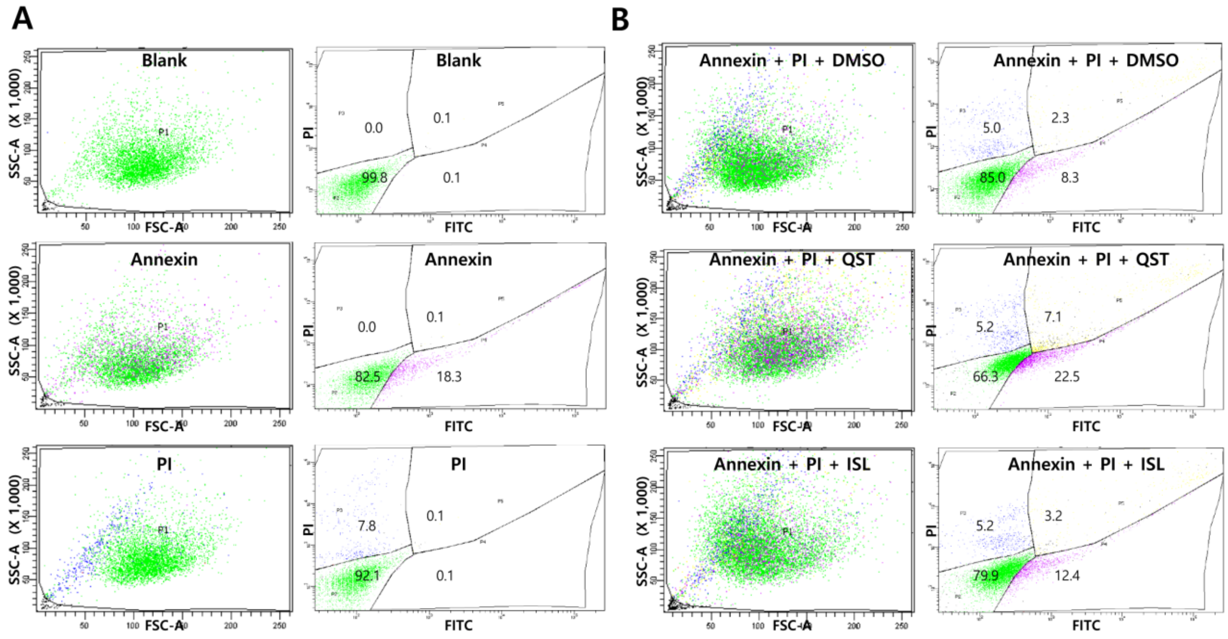

Supplemental Figure 1: Effects of quercetin or isoliquiritigenin on EBV infection. To analyze effects of quercetin or isoliquiritigenin on apoptosis in SNU719, FITC-Annexin V Apoptosis Detection assay followed by FACS analysis was conducted. (A) As control experiment, SNU719 cells were single-strained with either AnnexinV or propidium iodide (PI) followed by FACS analysis. The percentage of cells stained in each quadrant is listed in the corner of each quadrant. Blank stands for no staining. Both Annexin and PI staining showed their specific signals in corresponding quadrants. (B) SNU719 cells treated with quercetin or isoliquiritigenin were double-stained with Annexin V and propidium iodide (PI) followed by flow cytometry analysis. Data are presented as a percentage of the cell population. The percentage of Annexin-V-positive cells was used to indicate apoptosis. Compared to DMSO treatment, quercetin treatment (62 μM) enhanced to induce early apoptosis and necrosis/late apoptosis in SNU719 cells, while isoliquiritigenin treatment (45 μM) did not show any difference. ISL and QST stands for isoliquiritigenin and quercetin, respectively.
